# Classification of Microarray Gene Expression Data Using an Infiltration Tactics Optimization (ITO) Algorithm

**DOI:** 10.3390/genes11070819

**Published:** 2020-07-18

**Authors:** Javed Zahoor, Kashif Zafar

**Affiliations:** Department of Computer Science, National University of Computer and Emerging Sciences (NUCES), Lahore 54000, Pakistan; kashif.zafar@nu.edu.pk

**Keywords:** infiltration tactics optimization algorithm, classification, clustering, cancer, microarray, ensembles, machine learning, infiltration, computational intelligence

## Abstract

A number of different feature selection and classification techniques have been proposed in literature including parameter-free and parameter-based algorithms. The former are quick but may result in local maxima while the latter use dataset-specific parameter-tuning for higher accuracy. However, higher accuracy may not necessarily mean higher reliability of the model. Thus, generalized optimization is still a challenge open for further research. This paper presents a warzone inspired “infiltration tactics” based optimization algorithm (ITO)—not to be confused with the ITO algorithm based on the Itõ Process in the field of Stochastic calculus. The proposed ITO algorithm combines parameter-free and parameter-based classifiers to produce a high-accuracy-high-reliability (HAHR) binary classifier. The algorithm produces results in two phases: (i) Lightweight Infantry Group (LIG) converges quickly to find non-local maxima and produces comparable results (i.e., 70 to 88% accuracy) (ii) Followup Team (FT) uses advanced tuning to enhance the baseline performance (i.e., 75 to 99%). Every soldier of the ITO army is a base model with its own independently chosen Subset selection method, pre-processing, and validation methods and classifier. The successful soldiers are combined through heterogeneous ensembles for optimal results. The proposed approach addresses a data scarcity problem, is flexible to the choice of heterogeneous base classifiers, and is able to produce HAHR models comparable to the established MAQC-II results.

## 1. Introduction

Microarray experiments produce a huge amount of gene-expression data from a single sample. The ratio of number of genes (features) to the number of patients (samples) is very skewed which results in the well-known curse-of-dimensionality problem [1]. This further imposes two self-inflicting limitations on any proposed model: (i) processing all the data is not always feasible; and (ii) processing only a subset of data may result in loss of information, overfitting, and local maxima. These two limitations directly impact the accuracy and reliability of any machine learning model. To address the curse-of-dimensionality, a lot of research has been done in the past to identify the most impactful feature subset [2,3,4,5]. Both evolutionary as well as statistical methods have been proposed in the literature for this purpose. Feature Subset Selection (FSS) techniques like Minimum Redundancy Maximum Relevance (mRMR), Joint Mutual Information (JMI), and Joint Mutual Information Maximization (JMIM) are amongst the most prominent statistical methods [6,7,8] while advanced approaches like Particle Swarm Optimization (PSO), Genetic Algorithm (GA), Deep Neural Networks (DNN), Transfer Learning, mining techniques, etc. have also been shown in the literature to produce highly accurate results [9,10,11]. The microarray data classification process is typically carried out in two major phases: (i) Feature Selection: this phase focuses on selecting the most relevant features from otherwise a huge dataset to reduce noise, computational overheads, and overfitting. (ii) Classifier Training: this phase builds a model from the selected features to classify a given microarray sample accurately and reliably [12]. Advanced techniques like Deep Neural Network (DNN), Convolutional Neural Network (CNN), Transfer learning, Image processing, ANT Miner, and other exploratory approaches have been proposed in the literature [13,14,15,16,17,18,19,20,21]. While the advanced approaches for both FSS and Classifier training are capable of producing high accuracies, they need to be tuned according to the underlying dataset in a controlled setup to achieve these good results. However, in practice, there are a number of factors that can impact the accuracy and reliability of a model. These include the different cancer types that need analysis of different tissues, the differences in microarray toolkits/hardware e.g., data ranges and durabilities, experimental setups, number of samples, number of features used, type of preprocessing methods applied, validation method used, etc. Due to these variations and No Free Lunch (NFL) theorem, many of the existing methods can not be generalized across datasets. Thus, it is still a challenging problem for researchers to develop a generalized approach that can enhance both the reliability and accuracy of the model across datasets and variations. The algorithm proposed in this paper puts these variations at an advantage by using ensembles for the classification of microarray gene expression data. The Infiltration Tactics Optimization (ITO) algorithm proposed in this paper is inspired by classic war-zone tactics [22]—not to be confused with the ITO algorithm based on the Itõ Process in the field of Stochastic calculus [23,24]. It is comprised of four phases: Find, Fix, Flank/Fight, and Finish i.e., the so called Four F’s of the basic war strategy. A small light-infantry group (LIG) penetrates into the enemy areas to setup a quick command and control center while the follow-up troops (FT) launch a detailed offensive with heavier and sophisticated weapons to gain finer control and victory over the enemy. Both the LIG and FT members independently identify enemy weak-points and choose their own routes, targets, movements, and methods of attack. The “successful” LIG members are then combined to form a heterogeneous group that can become operational in a short time-interval. This LIG group is joined by the “successful” survivors from the FT to gain full control. The following text describes the four Fs (i.e., Find, Fix, Flank/Fight and Finish stages):

1. **Find:** In this stage, the LIG members analyze the field position to make a strategy and find the most appropriate target to attack.
2. **Fix:** In this stage, the LIG members use different light-weight weapons to infiltrate into enemy areas.
3. **Flank/Fight:** In this stage, LIG members keep the enemy pinned down so they could not reorganize their forces while the FT performs a detailed offensive in the area independently.
4. **Finish:** In this stage, the FT members apply heavier weapons to cleanup the area and gain full control over the enemy.

The proposed ITO algorithm is inspired by the Super Learner algorithm [25] but works in two phases to build the overall model. In the first phase, ITO builds a heterogeneous ensemble of parameter-free classifiers which can produce comparable results in a very short time-span. This sets a bar for the minimum accuracy and reliability of the overall ensemble which is further refined when fully tuned parameterized classifiers are available. The final model is guaranteed to meet this bar for accuracy and reliability at the minimum. Parameter tuning is generally very time-consuming and mostly it produces the most optimal results.

The microarray technology produces thousands of gene expressions in a single experiment. However, the number of samples/patients is much smaller (upto few hundreds) as compared to the number of features (several thousands). The small number of samples (training data) are not sufficient to build an efficient model from the available data. This is known as data scarcity in the field of machine learning. The ITO algorithm overcomes the data scarcity problem by building multiple heterogeneous base classifiers. ITO does not restrict the use of any base classifiers as LIG and/or FT members. It is possible to use the most performant classifiers from literature with this algorithm. The LIG and FT use exploration to learn about the different configurations and gain knowledge about rewards while the ensembling phase exploits the best performers from both LIG and FT to build an optimal model. The ITO algorithm achieves generalization and reliability by addressing data scarcity problems and producing HAHR models.

The rest of the paper is organized as follows; Section 2 provides a background of microarray-based cancer classification domain and literature review, Section 3 presents the proposed algorithm, Section 4 describes the experimental setup. Section 5 discusses the results and analysis and Section 9 presents the conclusions and future directions.

## 2. Background and Literature Review

Microarray gene expression data processing is a multidisciplinary area of computer science spanning graph analysis, machine learning, clustering, and classification [13]. Microarray technology allows measuring several thousand gene expressions in a single experiment. Gene expression levels help determine correlated genes and disease progression, which in turn helps in early diagnosis and prognosis of different types of cancers.

### 2.1. Phases of Microarray Gene Expression Data

#### 2.1.1. Phase 1: Pre-Processing

First of all, the gene expression data are discretized for noise reduction, missing values are imputed, and the data are normalized [13].

#### 2.1.2. Phase 2: Feature Subset Selection

Feature subset selection (FSS) helps in reducing the width of dataset which is skewed due to a very high features-to-samples ratio. A feature subset is selected such as to reduce feature redundancy without loss of information. There are generally three approaches for feature subset selection: i.e., filtering, wrapper based, or hybrid. Filtering approaches include minimum Redundancy and Maximum Relevance (mRMR), Mutual Information (MI), Joint Mutual Information (JMI), Joint Mutual Information Maximization (JMIM), etc. [4,5,8]. They perform feature selection without any information about the downstream classifier to be used. Thus, the feature selection is independent of classification. Wrapper-based approaches result in higher accuracy but are computationally expensive because they use an embedded classifier to gauge their performance [4,5]. A hybrid approach makes use of a combination of both filtering and wrappers [4,5], but the classifier used during feature selection may be different from the downstream classifier used for actual classification.

#### 2.1.3. Phase 3: Learning and Classification

In this phase, generally supervised classifiers are used with a subset of feature to train the model. Different techniques are used for two-class and multi-class classification. State of the art includes advanced techniques like transfer-learning, deep learning, convolutional neural networks, etc. or swarm optimization techniques like Ant Colony Optimization (ACO), Bat algorithm (BA), etc. However, overfitting (due to few training samples) and no-free-lunch theorem (NFL) (due to variations in underlying Microarray technology, cancer subtypes and different cancers resulting in different expressive genes, etc.) still remain two major challenges for most of the machine learning based techniques [9,26]. This research covers two-class problem only and uses ensemble of heterogeneous base classifiers to overcome overfitting and data scarcity.

### 2.2. Literature Review

#### 2.2.1. Microarray Quality Control (MAQC)

MAQC was a series of studies to monitor and standardize the common practices for development and validation of microarray based predictive models. The first phase of this project focused on addressing inter and intra-platform inconsistencies of results produced by different alternatives and methods. The aim was to setup guidelines for reproducible results in different setups using different hardware [27,28,29]. MAQC-II was the second phase of this project which aimed to establish a baseline for microarray gene expression data analysis practices. The purpose of establishing this baseline was to assess the reliability of clinical and pre-clinical predictions made through different models. For this purpose, 36 independent teams analyzed six microarray datasets with respect to 13 end points indicative of lung or liver toxicity in rodents or of breast cancer, multiple myeloma or neuroblastoma in humans. More than 30,000 different models were produced by these teams using different alternatives of analysis methods. MAQC-II used Matthews Correlation Coefficient (MCC) as the primary metric to evaluate the models [12]. MCC is used as a measure of quality for two-class classification. It ranges between [−1, 1] interval with MCC = 1 representing perfect prediction, MCC = 0 representing random predictions and MCC = −1 representing completely −ve correlation between the predictions and actual classes. MCC works better than other measures such as F-Score for microarray data with unbalanced class distribution [30]. The subsequent phase of MAQC (SEQC/MAQC-III) was focused on quality control for RNA Sequencing technologies rather than Microarray technology [31]. The MAQC-II established baseline results are thus taken up in this study to compare our results against using MCC as a primary metric. However, very limited research have reported results in the form of MCC.

#### 2.2.2. Feature Selection Algorithms

In 2005, Ding et al. proposed the famous minimum Redundancy and Maximum Relevance (mRMR) technique which made it possible to identify most relevant genes that can be used to reduce computational cost while maintaining high accuracy [6]. It uses mutual information with the target classes to determine relevant of a feature and dissimilarity of a selected feature with the already selected features. Since it computes both relevance and dependency independently, it is very likely that it may miss out a feature that individually looks irrelevant but when used in combination with other features may become significant i.e., it may miss out on interdependence of the features.

In 2014, Nguyen et al. analyzed the Mutual Information (MI) based approaches and contended that most of them are greedy in nature, thus are prone to sub-optimal results. They proposed that the performance can be improved by utilizing MI systematically to attain global optimization. They also reviewed the Quadratic Programming Feature Selection (QPFS) in detail and pointed out several discrepancies in QPFS regarding self-redundancy. They proposed spectral relaxation and semi-definite programming to solve this global optimization problem for mutual information-based feature selection. Their experiments show that spectral relaxation approach returns a solution identical to semi-definite programming approach but at a much lesser cost [32]. In addition, the spectral relaxation reduced the computation time to $O(n^{2})$ equivalent to mRMR. They also demonstrated empirically that their proposed method was much more scalable as compared to other methods in terms of computational time needed and working memory requirements. However, the computational time for mRMR with careful optimization was much better than their proposed global method.

In 2019, Potharaju et al. introduced a novel distributed feature selection method to remedy the curse-of-dimensionality of microarray data [33]. Their technique is inspired by an academic method of forming final year project groups. They used Symmetrical Uncertainty, Information Gain, and Entropy to build multiple balanced feature clusters. Each cluster is used to train a multi-layer perceptrons (MLP) and the most performant cluster is chosen for further processing. The MLP training and tuning itself is a very time-consuming task. Training multiple such clusters makes it even more resource hungry. However, the use of MLP makes it possible to stop the process prematurely and pick up the cluster with the highest accuracy and lowest root mean square for further processing. This approach may not scale well for a very large number of features because of computational and working memory requirements. It will further require a way to strike a balance between the cluster size and number of clustered required for such large datasets.

#### 2.2.3. Ensemble Based Approaches

In 2006, Wang et al. used Neuro-Fuzzy Ensemble (NFE) approach to utilize many inputs by dividing them into small (not necessarily disjoint) subsets that were used as input to individual Neuro-fuzzy units to learn a model. The outputs of these units were then used in an ensemble to jointly estimate the class of a sample. This approach made the biological interpretation of the selected features more sensible [34]. However, this approach requires encoding of prior knowledge from different sources, interpretation of the complete ensemble is still very complex, and it does not suggest how to balance between accuracy and use of existing knowledge for interpretability. These problems also make it hard to scale.

In 2009, Chen et al. proposed and showed that Artificial Neural Network (ANN) Ensemble with Sample Filtering is more accurate and stable than single neural network. This method also outperformed Bagging, Filtering, SVM, and Back Propagation [35]. However, the homogeneous ensemble of ANN requires a lot of computational time and resources to train each of the base ANN, thus it is not scalable for datasets with a very large number of features.

In 2013, Bosio used biological knowledge e.g., gene activation from Gene Ontology databases and statistical methods to generate meta-genes. Each meta-gene represented the common attributes of the contributing genes, thus replacing a number of genes with a representative meta-gene that yields better accuracy. He used Improved Sequential Floating Forward Selection (IFFS) and meta-genes to consistently out perform other models from literature [36]. However, this approach is mostly brute-force i.e., it needs to compute all pairwise correlations to generate meta genes which are also treated as genes/features for further processing. Although the IFFS algorithm eventually generates very small feature subset comprising of both meta-genes and raw genes where meta-genes represent cluster/tree-let of genes; however, based on the iterative nature of IFFS algorithm at each step, it chooses the best gene from amongst all genes and checks if adding increases the model performance; if not, then it checks if any existing genes should be removed or replaced with some other genes to make the feature subset the most optimal one. This makes the overall feature selection step very time consuming. Thus, scaling this approach for larger datasets will be a challenge.

#### 2.2.4. Heterogeneous Ensemble Classifiers

In 2008, Gashler et al. showed that a heterogeneous ensemble of different tree algorithms performs better than homogeneous forest algorithms when the data contain noise or redundant attributes [37]. This is particularly suitable for microarray data which contains a huge number of features, some of which could be a mere noise and other noise introduced during data digitization from the microarray chip. They use Entropy reducing Decision Trees and a novel Mean Margin Decision Trees (MMDT) to build the heterogeneous ensemble. Their work also showed that a small heterogeneous ensemble performs better than relatively larger homogeneous ensemble of trees. They used a diverse datasets comprising of many diseases, cars, wines, etc. to show how a heterogeneous ensemble can potentially address the NFL constrains. However, their work does not include MAQC-II Datasets and hence is not comparable with that benchmark.

In 2018, Yujue Wu proposed a Multi-label Super Leaner based on Heterogeneous ensembles to improve the classification accuracy of multi-class Super Learner [38]. A multi-label classification is a problem where each sample can represent more than one class labels simultaneously e.g., a picture may be assigned sea and beach or sea and mountains simultaneously depending upon the objects it contains. This work was not in the bio-informatics domain as such, but it was shown to outperform all other methods for music sentiment analysis, birds acoustics, and scenery datasets. Again, the diversity of problems it addresses shows the potential of heterogeneous ensemble to overcome NFL constrains.

In 2019, Yu et al. proposed a novel method using medical imaging, advanced machine learning algorithms, and Heterogeneous Ensembles to accurately predict diagnostically complex cases of cancer patients. They also used this system to explain what imaging features make them difficult to diagnose even with typical Computer-Aided Diagnosis (CAD) programs [39]. Their work takes lung images as input, performs segmentation of the image, and extracts features from them. These features are used to train the heterogeneous base classifiers and build an ensemble of trained classifiers. Their work improved the overall prediction accuracy to 88.90% as opposed to the highest accuracy reported in literature as 81.17%.

#### 2.2.5. Bio-Inspired Algorithms

In 2011, a very detailed overview summarizing the overall research carried out in the literature was compiled by Elloumi et al. covering the challenges, solutions, and future directions for Bio-Inspired algorithms [40].

In 2014, Selvaraj et al. compiled a list of applications of modern bio-inspired algorithms. Some of these algorithms have been applied to cancer detection already. These algorithms can be applied to microarray gene expression data to resolve the complex optimization problems posed by this data [14].

In 2016, Mohapatra et al. used modified Cat Swarm Optimization algorithm for feature selection along with Kernel Ridge Regression (KRR) for classification. They demonstrated that KRR outperforms wavelet kernel ridge regression (WKRR) and radial basis kernel ridge regression (RKRR), irrespective of the dataset used. Their technique performs relatively better on two-class datasets as opposed to multi-class datasets [41].

#### 2.2.6. Deep Learning Based Approaches

In 2013, Rasool et al. used Deep Learning based unsupervised feature learning technique and microarray data to detect cancer. PCA was used for dimensionality reduction. PCA along with a random subset of features (to ensure that nonlinear relations amongst the features are not completely lost due to PCA) are fed to auto-encoders to learn the gene-expression profiles. These gene-expression profiles are compared with healthy tissues’ profiles to detect the disease. This approach generalizes the feature subsets across different cancer subtypes. Their proposed method combines data from different tissues (cancer types and subtypes) to train the classifier for type-agnostic cancer detection. Thus, addressing data scarcity problem as well [9]. However, they did not use MAQC-II datasets in their study. They claim their approach to be scalable across cancer types and bigger datasets. However, because of missing time complexity analysis, missing parameter details of DNN, and very high level description of steps, this claim can not be validated.

In 2016, Chen et al. proposed a deep learning based model code-named D-GEX to infer the gene expression-levels of correlated genes based on the “landmark” genes. The idea of landmark genes suggests that carefully selected 1000 genes can help infer 80% of the genome-wide gene expression levels [10]. They trained their system using Microarray Omnibus dataset (not used MAQC-II datasets) This idea can be used as a pre-processing step to impute missing values for microarray data. The proposed model in this paper was compared with Linear Regression based current model and KNN based models and shown to outperform both of the. However, the interpretation of the learned hidden layers was found to be extremely difficult due to the complex way DNNs work i.e., lots of weights and nodes representing learned hidden structures from data. In addition, their implementation used random splitting of genes into smaller clusters due to hardware limitations. In its current state, this model is not scalable. However, as proposed in the paper, with the help of gene expression profiles, related genes could be clustered together and dimensionality reduction could be applied at a cluster level before processing them with DNN. This can greatly simplify the hidden structure that the DNN needs to learn and hence reduce computational needs for DNN.

In 2019, Liao et al. presented a novel Multi-task Deep Learning (MTDL) method that can reliably predict rare cancer types by exploiting cross cancer gene-expression profiling [21]. They used different datasets one for each type of cancer and common hidden layers that are extracted from these datasets to train the model. The trained model’s learning is then transferred as additional input to the prediction model. Their work showed significant improvement in correct diagnosis when there is inadequate data available. The performance improvements were evident in all but the Leukemia database where multi-class data are used. The proposed model learns common features from 12 different types of cancers to effectively exploit the right features for a given cancer type. Their work also showed the way to generalize a model across cancer-type and across datasets. The simplified approach of combining single task learners through a DNN and use of Transfer learning makes it a scalable model for two-class problems. For multi-class problems, further improvement will need to be done.

#### 2.2.7. Image Based Cancer Classification

In 2016, Huynh et al. extracted tumor information from mammograms to train their SVM classifier for cancer detection. They showed that the image-features learnt from mammograms performed comparable to the analytical feature selection methods [17]. A separate study by Spanhol et al. in 2016 used patches of histopathological breast images from BreaKHis database with CNN to classify samples for breast cancer. They used simple fusion rules to improve the recognition rates. Their final results outperformed the other results reported in the literature [18]. In another study in the same year, L’evy et al. used pre-segmented mammograms with Convolutional Neural Networks to measure breast-mass for binary cancer classification. Their method surpassed the expert human performance [20].

In 2017, Han et al. used histopathological breast images in conjunction with Deep Convolution Neural Networks (DCNN) to achieve automated cancer multi-class classification (subtype detection). Their proposed method achieved over 93% accuracy over a large-scale dataset BreaKHis. Employing Class-Structure aware approach (hence the name CSDCNN), they used oversampling over the training dataset to balance the class distributions amongst unbalanced classes. They also showed that the performance of their proposed method was significantly better with transfer learning (from an Imagenet dataset fine-tuned on the BreaKHis dataset) than learning the model from scratch directly on BreaKHis. Their work was the first attempt at Image based classification of Breast Cancers [19].

In 2020, Duncan et al. compiled a set of the ten most recent contributions in the fields of Big-Data, Machine Learning, and Image analysis in the Biomedical field and set the stage for upcoming cross-cutting concerns in these three areas [15].

#### 2.2.8. Cancer Detection Using Transfer Learning

In 2016, Huynh et al. used transfer learning from a deep CNN to learn tumor information (features) from the mammograms. These features were used with SVM to classify cancerous samples. They showed that this approach produced comparable results to the conventional FSS techniques. Furthermore, they formed an ensemble to achieve an accuracy higher than these two methods [17].

In 2017, Ravishankar et al. studied the process of transferring a CNN trained on ImageNet for general image classification to kidney detection problem in ultrasound images. They proved that transfer learning can outperform any state-of-the-art feature selection pipeline [42]. They further proved that a hybrid approach can increase the accuracy by 20%.

In 2018, transfer learning with Deep Neural Networks was used on unsupervised data from other tumor types to learn the salient features of a certain type of cancer. They tested their approach on 36 binary benchmark datasets from GEMLeR repository to prove that their approach outperformed many of the general cancer classification approaches [11].

The use of datasets for other cancer types and use of Transfer Learning makes these approaches scalable and worthy for further investigation. The effectiveness of their approach should be tested on MAQC-II benchmark datasets to gauge their reliability.

#### 2.2.9. Summary of Literature Review

Based on the advanced techniques presented in literature review, most of the studies have reported comparable results in terms of accuracy and reliability. However, not all of the studies are based on MAQC-II datasets and they use different scoring metrics like T-test, chi-test, MCC, error rate, confusion matrix, etc. Therefore, they cannot be benchmarked uniformly and compared on a common ground.

## 3. Proposed Algorithm

The proposed algorithm is inspired by warzone tactics. It is comprised of the Four Fs (Find, Fix, Flank/Fight, and Finish) of basic war strategy for infiltration into enemy areas i.e., small light-infantry group (LIG) backed by follow-up troops (FT) are used to conquer the area.

In our case, the LIG members are parameter-free classifiers that can be trained quickly to classify a sample with reasonable accuracy and reliability. The LIG members independently choose to identify enemy weak-points and choose their own routes, targets, movements, and methods of attack. While the overall approach does not restrict the user to use any particular classifiers and any set of parameter-free classifiers can be used; for this research, Decision Tree Classifier (DTC) [43], Adaptive Boosting (AdaBoost) [13,44,45,46] and Extra Tree Classifier (also known as Extremely Randomized Trees) [47] were used as LIG members with default settings.

The “successful” LIG members are then combined to form a heterogeneous ensemble which can reliably classify a given unseen sample. In parallel, the FT applies heavier and sophisticated techniques (i.e., parameter tuning) to find a better model. Random Forest [48], Deep Neural Network (DNN) a.k.a. Multi-layer Perceptron (MLP) [16,49] and Support Vector Machine (SVM) [50,51,52] were used as FT members with Grid Search and Random Grid Search for parameter tuning for binary classification. The “successful” FT members are used to update the overall ensemble for enhanced accuracy and reliability.

In the following text, we map the Four Fs (i.e., Find, Fix, Flank/Fight, and Finish stages) onto the proposed algorithm:

- **Find:** In this stage, a random grid search is applied on the 4-dimensional search space comprising of pre-processing methods, FSS methods, Subset sizes, and Validation methods to generate “attack vectors” (tuples of length 4 each from the search space with different combinations) for LIG and FT members e.g., (Quantile method, mRMR, 50 features, 10 Fold CV) is one such tuple. Details of the options used for each of these dimensions are given below.
- **Fix:** In this stage, each of the LIG members use one of the attack vectors to construct individual models. An efficiency index ρ is calculated using Matthews Correlation Coefficient (MCC) and average classification accuracy (score) as:
  (1) $$\rho_{LIG(i)}=MCC_{LIG(i)}\times score_{LIG(i)},$$
  where LIG(i) is the i-th member of LIG. Similar to MAQC-II benchmarks, MCC and accuracy are used to compute $\rho_{LIG}$. In addition, our analysis from earlier experimentation showed that, in the case of overfitting, though the average accuracy/score of the model seemingly improves but simultaneously the MCC of the model decreases. Hence, these two measures were used to decide the trade-off between accuracy and MCC at the time of base classifier selection. The value of MCC ranges between −1 and +1, but, for our experimentation, we used only (0, 1] or MCC > 0 i.e., anything better than random guess. The accuracy ranges between [0, 1] range. Both measures are equally important, thus we use product as a statistical conjunction function. It helps balance the trade-offs between MCC and Accuracy. In our experiments, we observed that $\rho_{LIG}$ helped in improving both the MCC and accuracy in some cases and helped achieve a good trade-off between MCC and accuracy in other cases. A fitness threshold $\epsilon_{LIG}$ is used to filter in “successful” members from the whole LIG i.e.,
  (2) $$\rho_{LIG(i)}>\epsilon_{LIG},1>\epsilon_{LIG}>0$$
  The value of $\epsilon_{LIG}$ is chosen such that it filters at least the top 33% of the LIG members for $LIG_{Ensemble}$. Once the ensemble is formed, the value of ϵ can be adjusted to tune the ensemble for maximum $\rho_{LIG-Ensemble}$ yield as explained below.
- **Flank/Fight:** In this stage, a heterogeneous ensemble of a subset of “successful” LIG members (MCC > 0) is formed such that:
  (3) $$\rho_{LIG-Ensemble}\geq \forall \rho_{LIG(i)}$$
  The ensemble is formed iteratively using a majority-vote method. In each iteration, the top LIG(i) is added to the $LIG_{Ensemble}$ and $\rho_{LIG-Ensemble}$ is computed to ensure that the newly added LIG(i) did not deteriorate the ensemble performance. If an LIG(i) causes decline in the $\rho_{LIG-Ensemble}$, it is discarded.
  The $LIG_{Ensemble}$ takes relatively very short time to build while each FT(i) may take several hours to days to train (depending upon the parameter-space), thus, for the time-sensitive cases e.g., in the domain of pandemic diseases where an early prediction may be required, $LIG_{Ensemble}$ can be used until FT(i) are being trained. When the FT(i) are trained and the ensemble updated for improved performance, a follow-up prediction could be done which will either strengthen the confidence in prediction if both $LIG_{Ensemble}$ and $Final_{Ensemble}$ agree on the prediction Or $Final_{Ensemble}$ could be used to over-ride the earlier prediction.
- **Finish:** In this stage, the FT members apply advanced classifiers such as Deep Neural Networks, SVM, etc. to build fine-tuned models. The “successful” FT members are filtered in using:
  (4) $$\rho_{FT(i)}=MCC_{FT(i)}\times score_{FT(i)}$$
  where FT(i) is the ith member of FT. A fitness threshold is used to filter in “successful” FT members i.e.,
  (5) $$\rho_{FT(i)}>\epsilon_{FT},1>\epsilon_{FT}>0$$
  Then, $FT_{Ensemble}$ is computed from FT subsets such that:
  (6) $$\rho_{FT-Ensemble}\geq \forall \rho_{FT(i)}$$
  Finally, a $Ensemble_{Final}$ is formed using filtered-in LIG(i) and filtered-in FT(i). The following different approaches can be used to build the $Ensemble_{Final}$:
  (a) simply combine all the LIG and FT members from $LIG_{Ensemble}$ & $FT_{Ensemble}$, respectively. However, through empirical analysis, it was found that this approach actually causes a decline in MCC and/or average accuracy of the model.
  (b) start with one of $LIG_{Ensemble}$ or $FT_{Ensemble}$ and call it $Ensemble_{Final}$. Choose base classifiers from the other ensemble with $\rho \geq \rho_{Final-Ensemble}$ and add to $Ensemble_{Final}$. However, starting with an ensemble with higher ρ would cause all of them to fail on $\rho \geq \rho_{Final-Ensemble}$, thus resulting in no further improvement. In addition, our experiments showed that, starting with an ensemble with lower ρ, the optimization gain was not as good as the next approach because the condition $\rho \geq \rho_{Final-Ensemble}$ filtered out many classifiers which still could help with reducing misclassifications of ensembles hence improve both the accuracy and MCC.
  (c) rebuild the $Ensemble_{Final}$ from scratch using LIG(i) ∪ FT(i) ordered by ρ. This approach was found effective to further enhance the performance.

While the proposed algorithm is flexible to allow the choice of any classifiers, the pre-processing method, validation method, subset size, and FSS methods, etc., the following configurations were used in this study for LIG and FT members to carry out the Four Fs.

The imputer method [51] was used for data normalization. During feature exclusion, the features with any missing values were completely removed from the dataset because (i) the number of features are in abundance already and, (ii) due to missing values, these features do not represent the sample space sufficiently. For scaling of the data Quantile method, Robust method, and Standard method were used [51].

While, in the most recent studies [53,54], multi-objective feature selection methods have been shown to outperform the single-objective methods; however, their implementations are not widely available for public use. Thus, for feature subset selection (FSS), also known as Variable Selection, three publicly available single-objective methods, namely Joint Mutual Information (JMI), Joint Mutual Information Maximization (JMIM), and minimum Redundancy Maximum Relevance (mRMR) were used [6,7,8,51]. The minimum number of features that should be chosen, largely depends upon the dataset being used. For the basic techniques like JMI and JMIM the produced subset may contain some level of redundancy whereas mRMR ensures that the chosen features in a subset have minimum redundancy and maximum relevance to the class label [6,8]. These are brute-force techniques and all the features are considered to compute a ranked list of features based on statistical relevance and hence it is a computationally expensive step [8]. The selection of these algorithms was done due to their out-of-the-box availability for Python, not requiring an implementation from scratch.

For validation, 10-Fold Cross Validation (CV) and Leave-one-out CV (LOOCV) were considered, both of which have been proven in the literature to be amongst the best validation techniques [36].

### Pseudo Code

The ITO Algorithm (Algorithm 1) computes $LIG_{Ensemble}$ using Algorithm 2. This produces an initial baseline result which is either the best of LIG members or an improved output from the ensemble. The Grid G on line 2 of Algorithm 1 has 4-tuples i.e., four elements wide and the length of G will be |preps| × |searchRadius| × |searchStrategy| × |successEvaluation| to hold all possible combinations of these four sets. The variables $t_{LIG}$ and $t_{LIG}$ (also 4-tuples) are subsets of G, for our experiments, we used half the size of G. The Compute$Ensemble_{Final}$ (Algorithm 3) conditionally updates this ensemble using a ranked list of FT(i) if they improve the overall results.
 **Algorithm 1:** ITO Algorithm**input**:  **T**: t × f matrix - training dataset with t samples and f features;  **V**: v × f matrix - validation dataset - with v samples and f features;  **preps**={Imputer, Robust, Quantile, Standard, …} - set of preprocessing methods;  **searchRadius**={10, 50, 100, 150, 200, 250, …} - set of FSS sizes;  **searchStrategy**={JMI, JMIM, mRMR …} - set of FSS methods;  **successEvaluation**={10 Fold CV, LOOCV, …} - set of validation methods;  $LIG_{Options}$={DT, AdaBoost, Extra Tree, …} - set of parameter-free classifiers;  $FT_{Options}$={DNN, SVM, Random Forest, …} - set of parameterized classifiers**output**: $Ensemble_{Final}$ **BEGIN** G ← GenerateOptionsGrid(searchRadius, searchStrategy, successEvaluation, preps); Choose $t_{LIG}$ ⊂ G using Randomized Grid Search; $LIG_{Ensemble}$ ← $ComputeLIG_{Ensemble}$ (T, V, $LIG_{Options}$, $t_{LIG}$) //Algorithm 2; Choose $t_{FT}$ ⊂ G using Randomized Grid Search; $Ensemble_{Final}$ ← $ComputeEnsemble_{Final}$ (T, V, $FT_{Options}$, $t_{FT}$) //Algorithm 3; **return**
$Ensemble_{Final}$; **END**

 **Algorithm 2:** Compute$LIG_{Ensemble}$

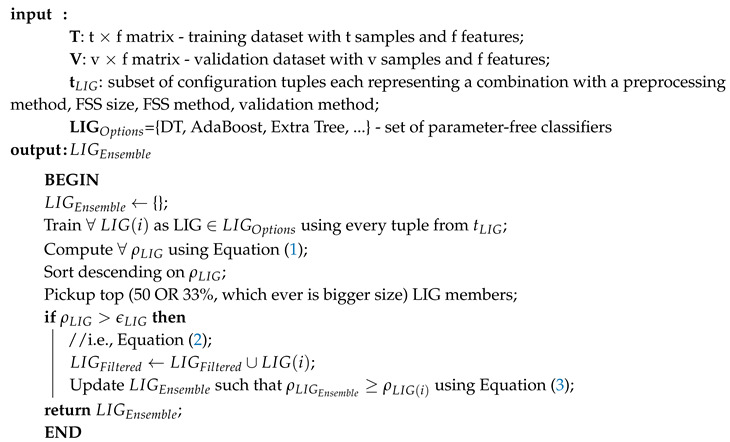



 **Algorithm 3:** Compute$Ensemble_{Final}$

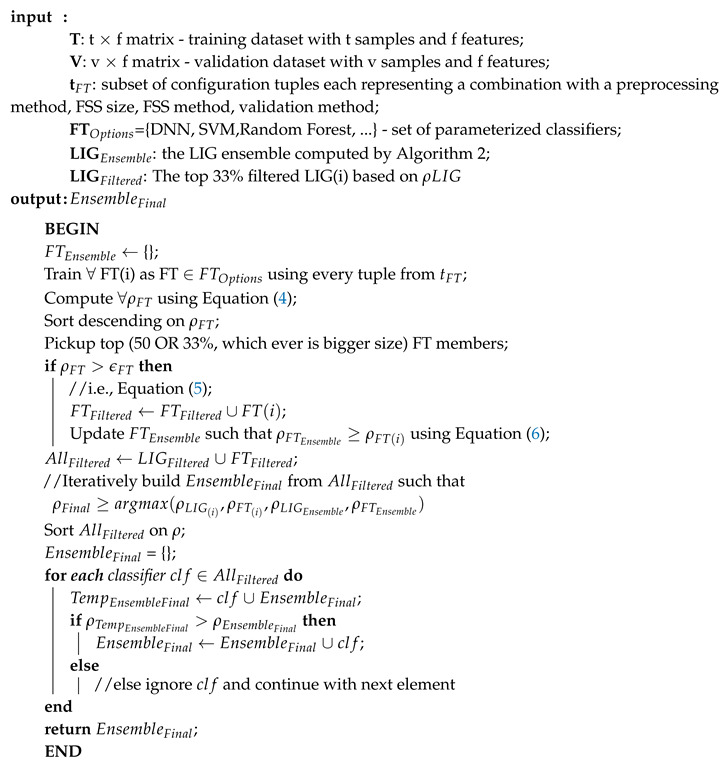



## 4. Experimental Setup

The ITO Algorithm was run on each of the Datasets A, B, and C to Compute $Ensemble_{Final}$ for each of the datasets, respectively.

### 4.1. Benchmark Datasets

There are a number of publicly available datasets that have been used in different research papers [13,40,55]. However, this research uses datasets A (Hamner), B (Iconix), and C (NIEHS) from Microarray Quality Control Study—Phase II (MAQC-II) [12] as listed in Table 1.

### 4.2. Description of Datasets Used

Dataset A (Hamner), Dataset B (Iconix), and Dataset C (NIEHS) from the MAQC II study have been used in this research. MAQC was a series of studies conducted by American Health Association (AHA) to establish a baseline for a reliable model for data classification Microarray data.

Dataset A (accession code GSE24061) was obtained from mice while conducting Lung tumorigen vs. non-tumorigen study using an Affymetrix Mouse 430 2.0 platform [12]. The training and validation datasets have 1,004,004 features (using raw .CEL files). The training dataset contains 26 positive samples and 44 negative samples (70 samples in total), while the validation dataset is comprised of 28 positive and 60 negative samples (88 samples).

Dataset B (accession code GSE24417) was data obtained for mice while conducting non-genotoxic liver carcinogens vs. non-carcinogens study. The separation of the training and validation set was based on the time when the microarray data were collected; i.e., microarrays processed earlier in the study were used as training and those processed later were used for validation. This study was conducted on an Amersham Uniset Rat 1 Bioarray platform. The training and validation datasets have 10,560 features (using GSE24417_Training_DataMatrix.txt.gz file). The training dataset contains 216 samples with 73 positive and 173 negative samples, while the validation dataset contains 57 positive and 144 negative samples (total 201 samples).

Dataset C (accession code GSE24363) was obtained from rat-liver while conducting liver necrosis prediction. The data was collected from 418 rats using an Affymetrix Rat 230 2.0 microarray. The training and validation datasets have 695,556 features (using raw .CEL files). The training dataset contains 214 total samples with 79 positive and 135 negative samples. The validation dataset contains 204 samples with 78 positive and 126 negative samples.

The experimental data used to support the findings of this study are available at https://github.com/JavedZahoor/phd-thesis-iv.

### 4.3. FT Parameter Setting

The objective of this research was not to find the most optimal parameters for the individual FT members but to demonstrate the effectiveness of the proposed algorithm over whatever base classifiers are used; hence, the best parameters found for FT members during these experiments are not guaranteed to be the most optimal ones. In addition, since, in the final run, 199 FT(i) were filtered-in for Dataset A based on ρ, 12 for Dataset B and 108 FT(i) for dataset C, thus, for the sake of brevity, the list of those 312 optimal parameters is skipped from the paper. However, for the interested readers, some details are provided here. The grid search was used on a small set of choices to find the best available setting for individual FT members. For DNN/MLP, the following parameter grid was used:

1. learning_rate: [“constant”, “invscaling”, “adaptive”]
2. alpha: [1, 0.1, 0.001, 0.0001, 0.00001, 0.000001, 0.0000001, 0.00000001]
3. activation: [“logistic”, “relu”, “tanh”]
4. hidden_layer_sizes: [(100,1), (100,2), (100,3)]

For SVM, the following parameter grid was used:

1. C = [0.001, 0.01, 0.1, 1, 10]
2. gamma = [0.001, 0.01, 0.1, 1]
3. kernels = [’rbf’,’linear’]

For the Random Forest, the following parameter grid was used:

1. estimators: [100, 300, 500, 800, 1000]
2. criterion: [‘gini’, ‘entropy’]
3. bootstrap: [True, False]

### 4.4. ITO Parameter Setting

The $\epsilon_{LIG}$ and $\epsilon_{FT}$ are used to control the number of classifiers that will be considered for $Ensemble_{LIG}$ and $Ensemble_{FT}$, respectively. These $\epsilon_{LIG}$ and $\epsilon_{FT}$ are set to control the number of LIG and FT members that are considered “successful”. This helps in preventing overcrowded ensembles. For this study, we numerically computed $\epsilon_{LIG}$ and $\epsilon_{FT}$ to ensure that the top 33% members for Datasets A & C are included for which hundreds of LIG and FT members produced MCC > 0. However, for Dataset B, where only few (less than 20) produced MCC > 0, we set the value of $\epsilon_{LIG}$ and $\epsilon_{FT}$ very low to allow all of them to be included. Table 2 summarizes the settings for $\epsilon_{LIG}$ and $\epsilon_{FT}$ that were used:

## 5. Results and Analysis

The ITO algorithm optimizes the overall model in two phases, LIG optimization and FT optimization.

### 5.1. LIG Optimizations

**Figure 1:**
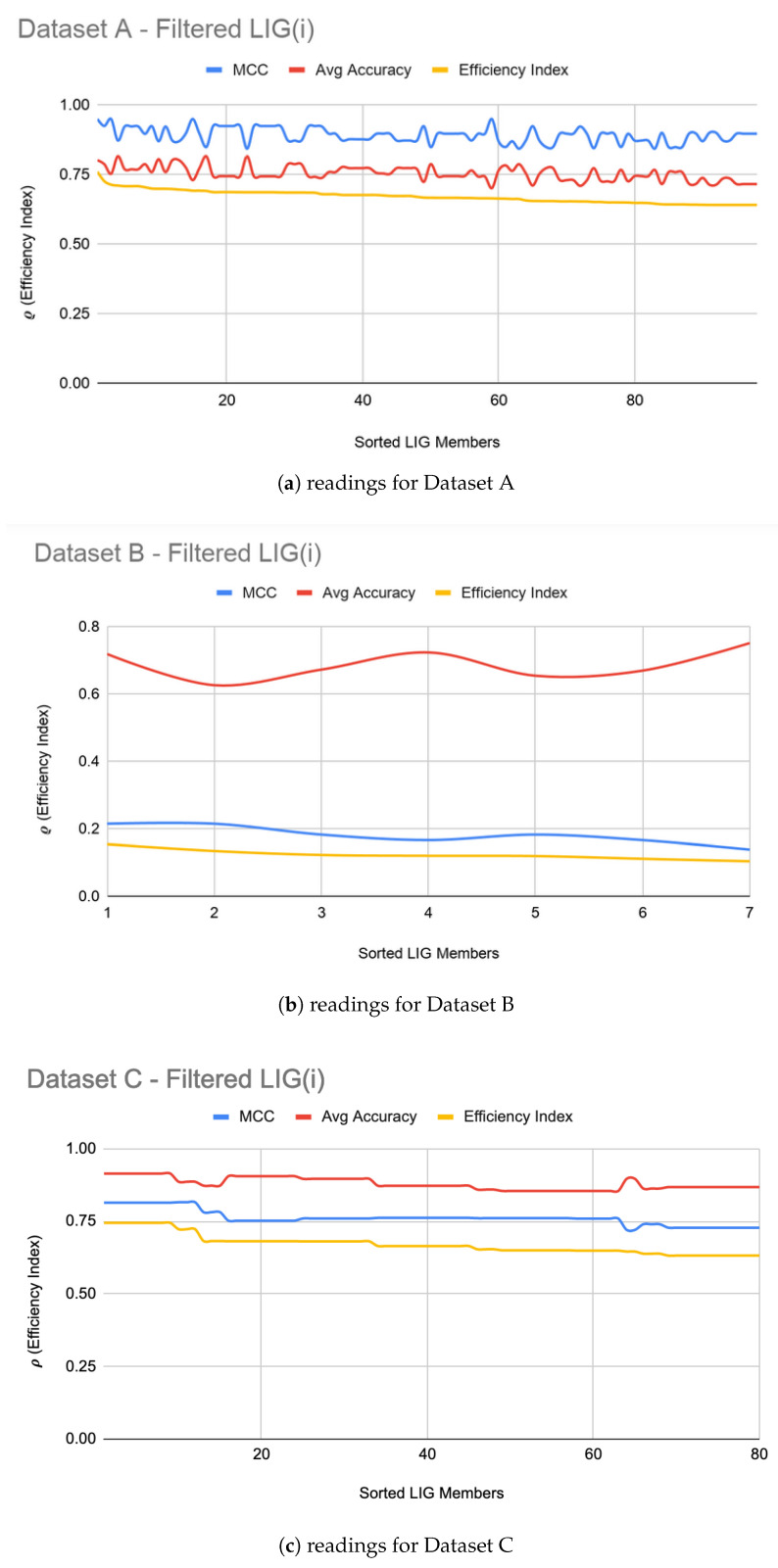
LIG(i) filtered-in accuracy, MCC, and ρ.

Figure 1a–c show the filtered LIG(i) of Datasets A, B, and C respectively sorted on their efficiency index (ρ). As it can be seen from Figure 1, selection of LIG members can not be done based on the MCC values or average accuracy alone, since they exhibit different and unrelated behavior for different LIG members. Thus, the efficiency index (ρ) is used as a fitness measure to rank LIG(i) from most efficient to least efficient. As a heuristic, only the top 33% of the successful (i.e., (ρ > 0) LIG(i) were filtered-in for further processing.

For Dataset A, $LIG_{Filtered}$ is comprised of 98 members. The $LIG_{Filtered}$ had MCC ranging in an 0.84–0.95 interval (avg 0.89) and the accuracy ranging in a 70–81% interval (avg 75%). For Dataset B, smaller subset sizes produced a high accuracy but poor MCC value hence the size of LIG group was only 7 members. The average accuracy of LIG(i) was found to be 63–75% (an average of 69%), whereas $LIG_{Ensemble}$ improved the average accuracy to 72%. However, due to poor MCC values in the range 0.14–0.21, the ensemble contained only one LIG member i.e., the highest performing member. For Dataset C, the chosen LIG group comprised of 80 LIG members and the LIG(i) average accuracy is 88% (in the range of 85–91%) with average MCC of 0.76 (in the range of 0.72–0.82).

**Figure 2:**
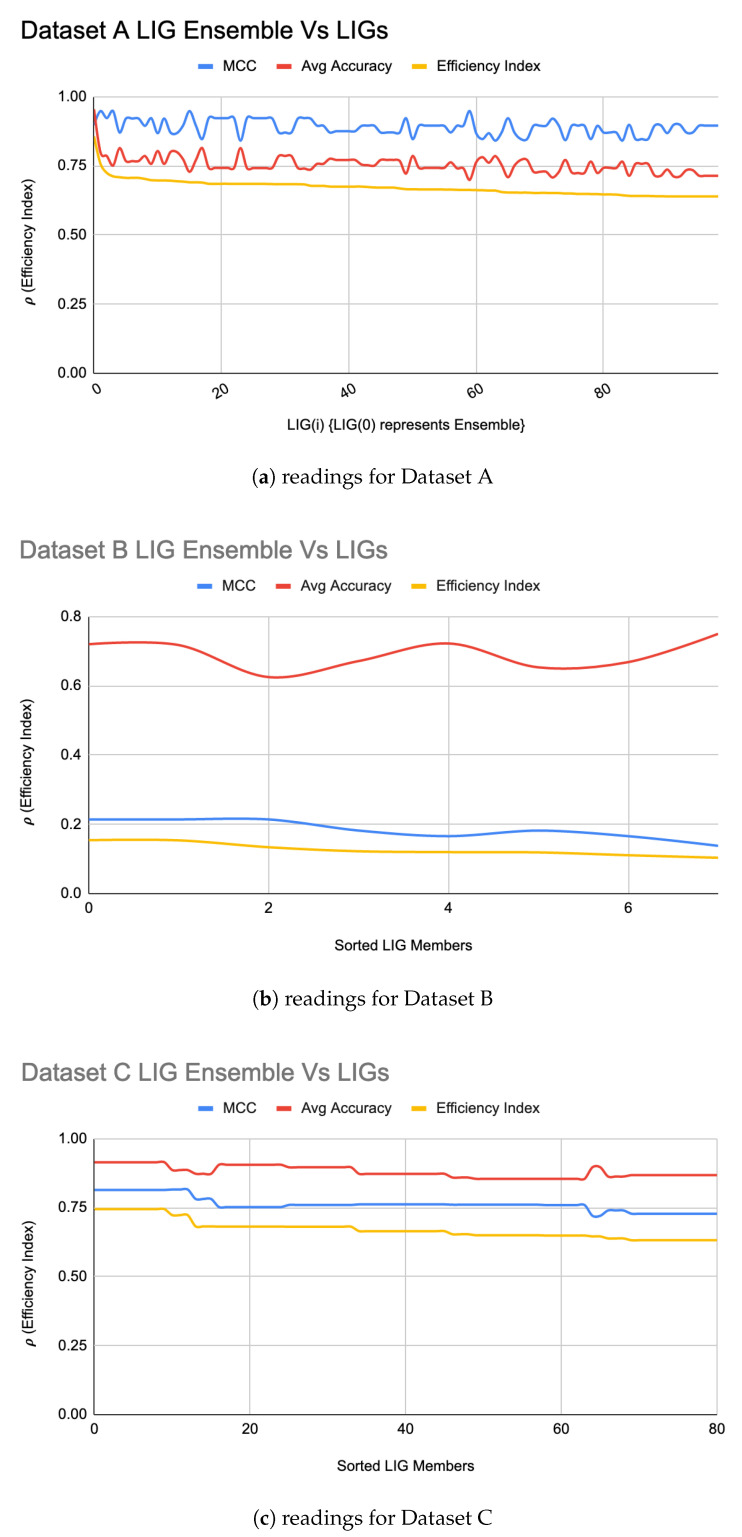
$LIG_{Ensemble}$ vs LIG(i) filtered-in accuracy, MCC, and ρ.

An $LIG_{Ensemble}$ was formed for each of the datasets using a majority-voting ensembling method to even out the individual biases of LIG(i). Figure 2a–c shows the efficiency index for $LIG_{Ensemble}$ (shown at i = 0 in the graph) for Datasets A, B, and C, respectively. For Dataset A, the $LIG_{Ensemble}$ resulted in a higher accuracy (95%) and MCC (0.90) as compared to the individual LIG(i) as shown in Figure 2a. For Dataset B, the accuracy improved to 72% with an improved MCC value of 0.21 as shown in Figure 2b. For Dataset C, the accuracy improved to 90% with an improved MCC of 0.82 as shown in Figure 2c. Table 3 summarizes the performance improvements through $LIG_{Ensemble}$:

Note that the value of ρ (Equation (1)) will always fall below the accuracy and MCC, but, due to its relative nature, the max value of ρ will always indicate the best LIG(i) (or FT(i)).

### 5.2. FT Optimizations

**Figure 3:**
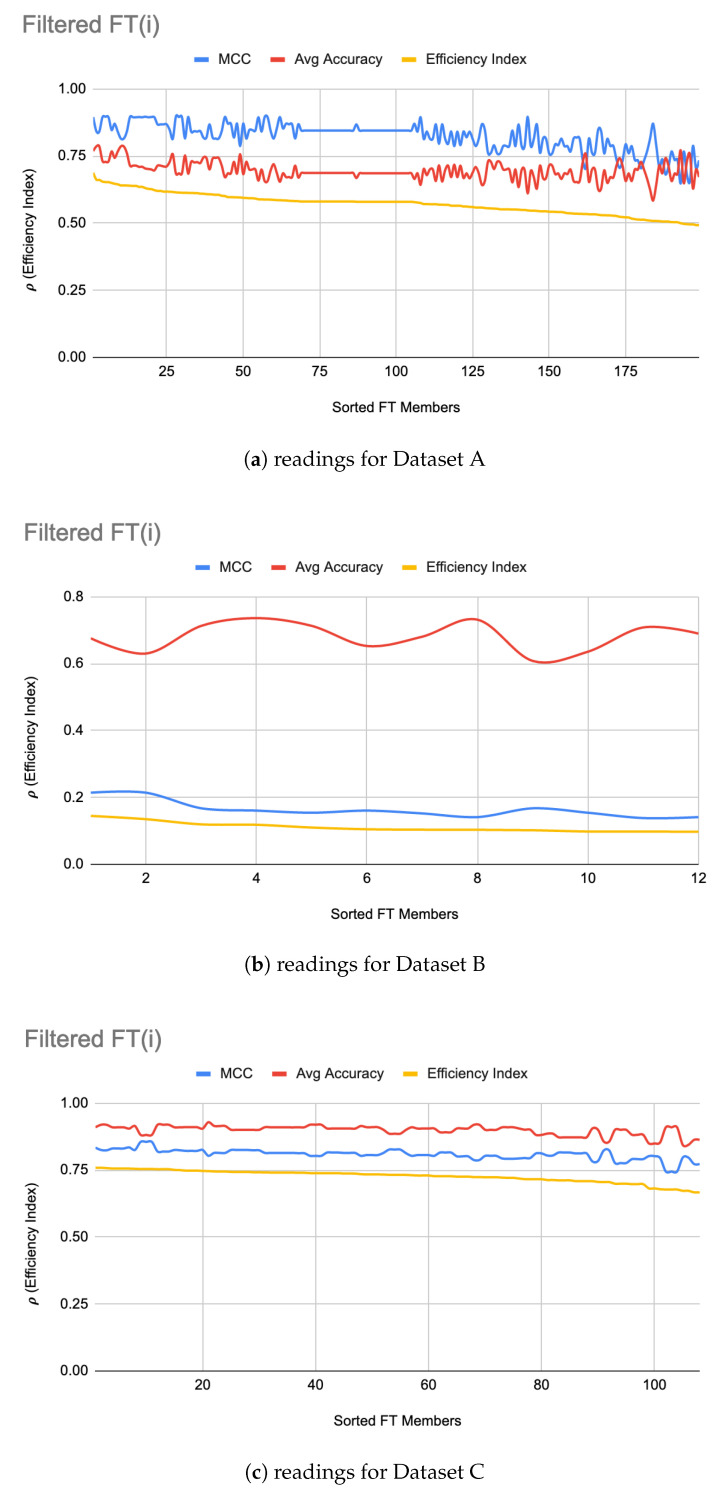
FT(i) filtered-in accuracy, MCC, and ρ.

As a next step, the FTs were trained on each dataset under the same configuration options except the set of classifiers, which, in this case, were parameterized classifiers, each requiring its own parameter tuning. Figure 3 shows filtered-in FT(i) and their ρ. For Dataset A, top 75 FT(i) were filtered-in based on their ρ. Similar to LIG(i) selection, as a heuristic, top 33% of “successful” FT(i) were chosen to construct the $FT_{Ensemble}$ which achieved an accuracy 97% as compared to average accuracy of 70% (ranging from 58–79%) and MCC to 0.92 as compared to average MCC of 0.82 (0.65–0.90). For Dataset C, the $FT_{Ensemble}$ improved the average accuracy to 91%, average MCC to 0.84 as shown in Figure 3c. For Dataset B, like before, only 12 members were chosen from FTs due to poor MCC values for all other members. The accuracies, MCC, and $\rho_{FT-Ensemble}$ of FT(i) can be seen in Figure 3b. Dataset B is a hard dataset to model [12], and it might be possible to get better individual results through the use of advanced base-classifiers such as CNN or PSO based implementations, etc. The limited choice of LIG and FT models used in this study (due to their out of box availability) did not produce higher MCC. Table 4 summarizes the performance improvements through $FT_{Ensemble}$:

ITO works independent of these choices, hence any better models can be used as a member for both LIG and FT. The proposed optimization method was still able to produce comparable overall accuracy and enhance the MCC value through optimization as shown in Figure 3c. Table 4 shows that, for dataset B, the ITO algorithm produced a HAHR model with comparable reliability and accuracy.

**Figure 4:**
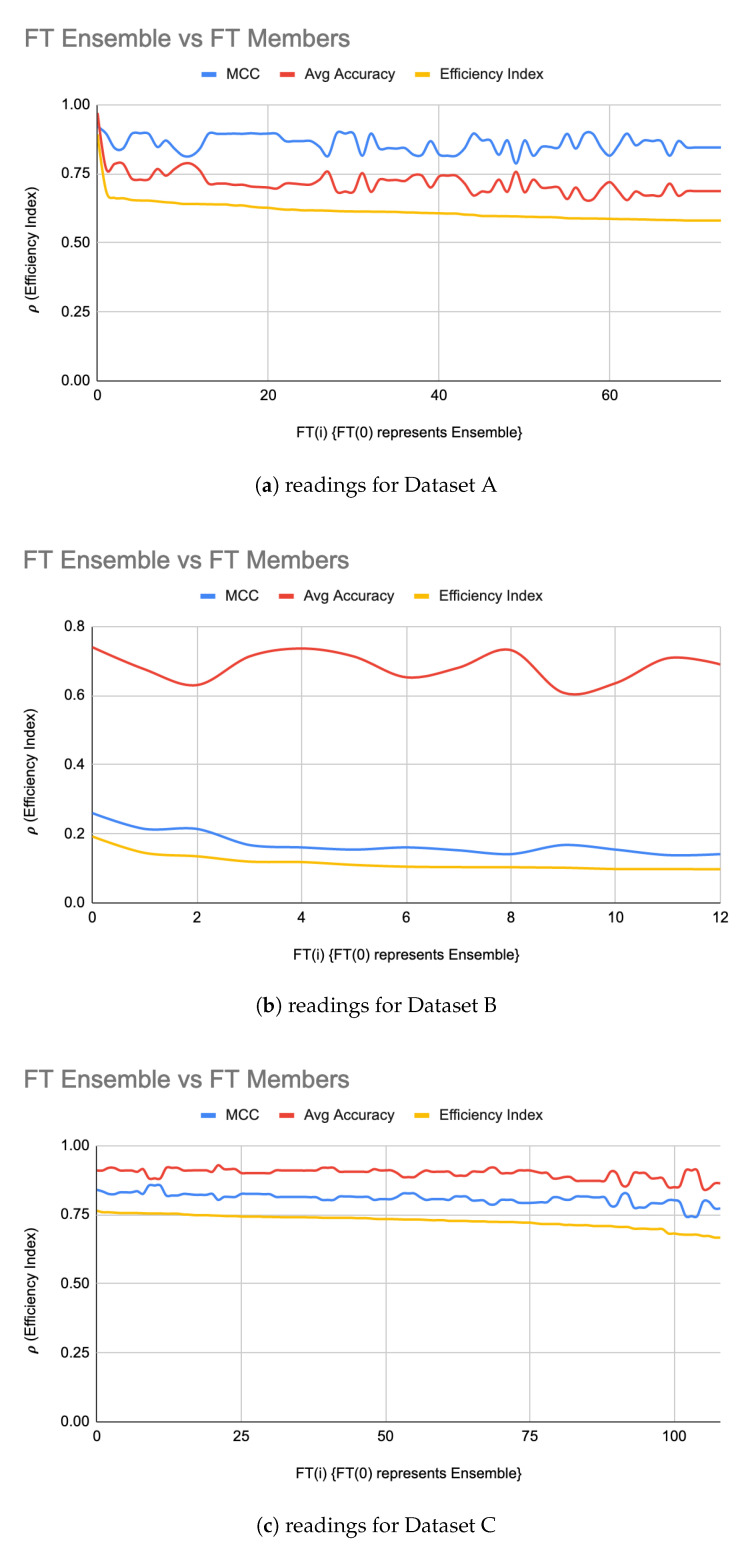
$FT_{Ensemble}$ vs. FT(i) filtered-in accuracy, MCC, and ρ.

From raw results, it was interesting to note that a noticeable majority of successful FT(i) were using RandomForest, followed by a relatively small number of FT(i) using SVM. $FT_{Ensemble}$ constructed from FT(i) resulted in a relatively very high efficiency index as shown in Figure 4a. It is interesting to note that, except for the first few LIG(i) and FT(i), the MCC values and average accuracies of the individual LIG(i) or FT(i) seemed to be inversely proportional to each other i.e., the higher accuracy, the lower reliability, and vice versa. This is a clear indication of over/under fitting of individual LIG(i) or FT(i).

**Figure 5:**
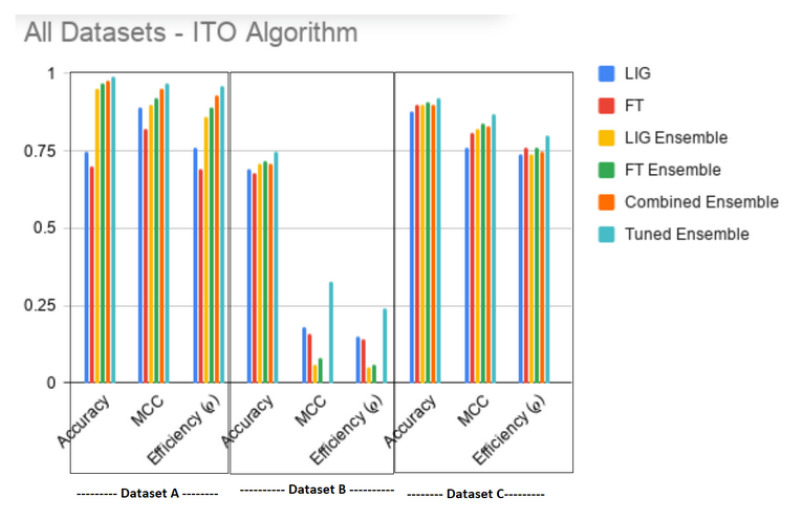
Overall improved results produced by ITO Algorithm on all datasets.

Finally, Figure 5 shows that the proposed algorithm produced an overall best result. It is interesting to note that, instead of choosing only $\rho_{FT(i)}>\rho_{LIG-Ensemble}$, updating the $LIG_{Ensemble}$ with top FT(i) without this constraint improved the $\rho_{LIG-Ensemble}$ i.e., $\rho_{overall-Ensemble}\geq argmax{(}_{LIG-Ensemble},\rho_{FT-Ensemble},\rho_{LIG(i)},\rho_{FT(i)})$. Table 5 and Table 6 show the values-of and %age improvement in MCC, Accuracy and ρ between ITO Tuned Ensemble against LIG(i), FT(i), $LIG_{Ensemble}$, $LIG_{Ensemble}$, and Combined Ensemble (i.e., an ensemble of all LIG(i) and FT(i)), respectively. Table 7 and Table 8 show that, for datasets A & C respectively, the ITO algorithm produced a HAHR model with significantly higher reliability and accuracy, whereas, for dataset B (Table 9, however, the accuracy increased, but the MCC decreased a bit.

Figure 5 and Table 5 and Table 6 show that ITO was able to enhance both the accuracy as well as MCC (and hence ρ) for all the datasets regardless of the base LIG and FT classifiers.

## 6. Machine Specifications

The experiments were performed on a shared machine with 64-bit ASUS GPU, 32 GB RAM, Quad-core 64-bit Intel i7-4790K CPU, with 800MHz-4.4 GHz speed.

## 7. Time Complexity

The overall time complexity of the algorithm depends on:

1. number of samples (t)
2. number of features (f)
3. number of different preprocessing methods (P) and maximum execution time for preprocessing ($t_{prep}$) of dataset of size txf
4. number of FSS methods (FM) and maximum execution time for feature selection ($t_{fss}$) from f features. This is one of the most time-consuming steps of the algorithm because the underlying methods need to calculate pair-wise mutual information for feature ranking, which is eventually used to pick the top features.
5. Subset sizes (S)
6. Validation methods (V)
7. number of parameterized classifiers ($C_{p}$) and maximum time to train a parameterized classifier ($t_{p}$) This is the second most time-consuming step of the algorithm. Parameter tuning for the classifiers requires trying different combinations of parameter values and find the most effective one.
8. number of non-parameterized (parameter-free) classifiers ($C_{n}$) and maximum time to train a parameter-free classifier ($t_{f}$).
9. Ensemble construction time ($E_{FT}$=FT Ensemble, $E_{LIG}$=LIG Ensemble, $E_{ITO}$=Overall Ensemble).

The time complexity of ITO algorithm would be as given in Equation (7).

(7) $$\left(P\times t_{prep}\right)\times \left(FM\times t_{fss}\right)\times S\times V\times \left(C_{p}\times t_{p}+C_{n}\times t_{f}\right)+E_{LIG}+E_{FT}+E_{ITO}$$

## 8. Execution Times

**FSS** was a very time-consuming step because, for the chosen methods, all pairwise correlations are computed between features to rank the most relevant features for final selection. To stay focused on the generalization problem, the FSS method was chosen solely considering the availability of out-of-the-box implementation or library for Python. Table 10 shows the minimum and maximum times it took to generate FSS for datasets A, B, and C.

**Figure 6:**
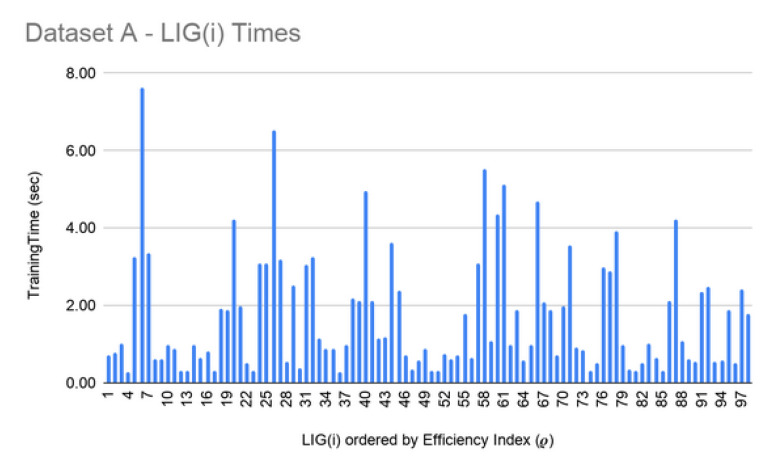
Dataset A—LIG(i) filtered-in training times.

**Figure 7:**
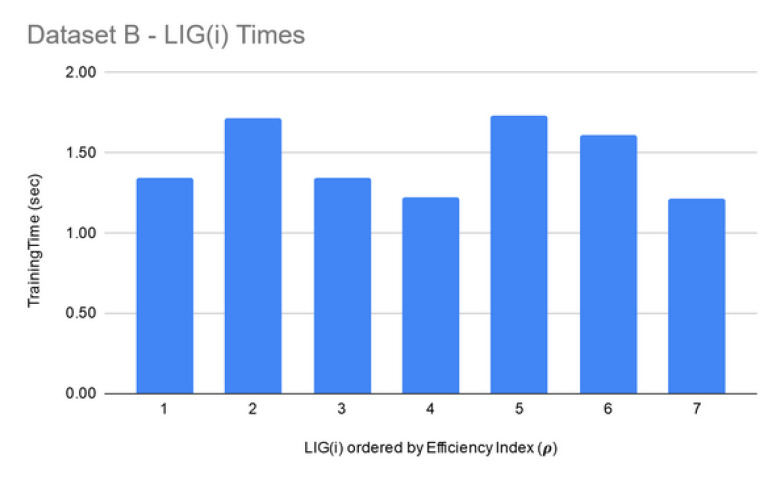
Dataset B—LIG(i) filtered-in training times.

**Figure 8:**
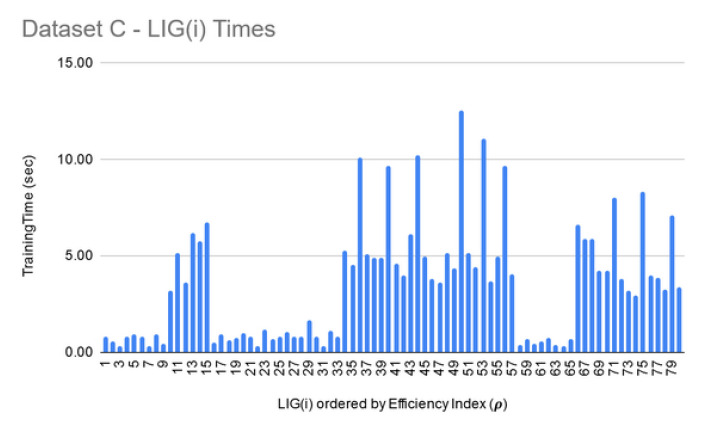
Dataset C—LIG(i) filtered-in training times.

**LIG training and filtering:** As can be seen from Figure 6, Figure 7 and Figure 8, the training time for the filtered-in LIG(i) was under 15 s each.

**LIG ensemble formation:** In this phase, an ensemble is formed iteratively using the majority-voting method. The execution time for this step was under 500 s.

**Figure 9:**
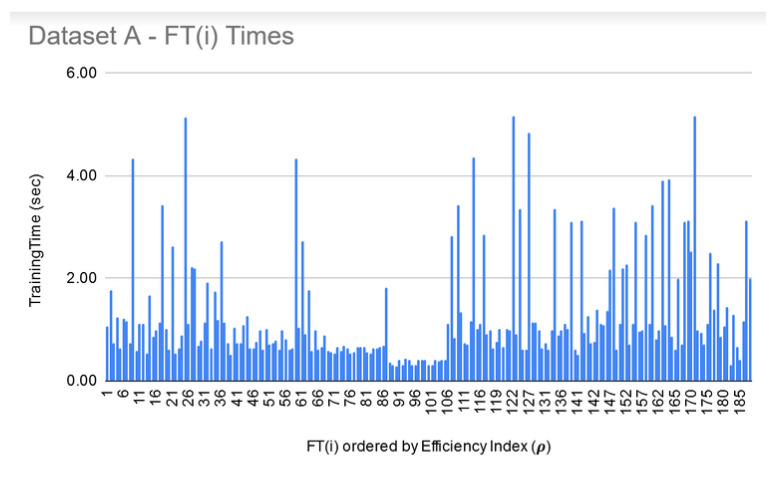
Dataset A—FT(i) filtered-in training times.

**Figure 10:**
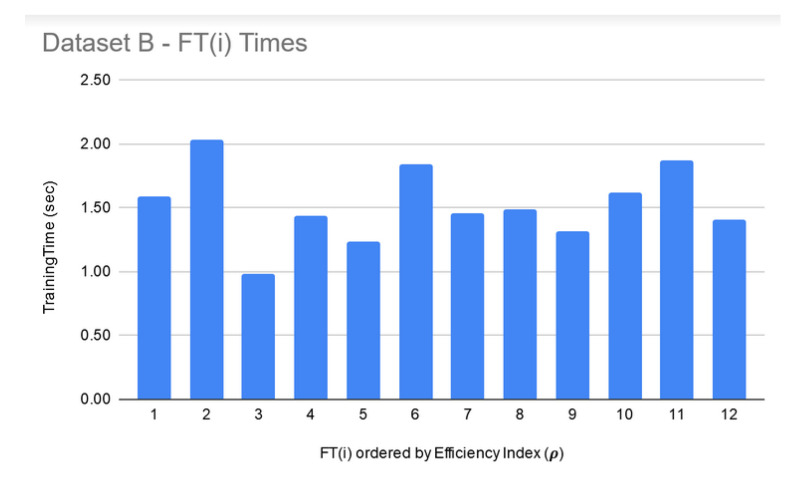
Dataset B—FT(i) filtered-in training times.

**Figure 11:**
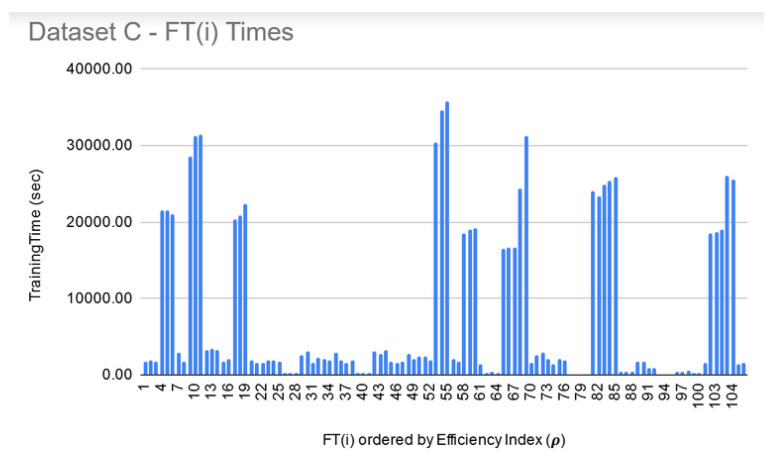
Dataset C—FT(i) filtered-in training times.

**FT training and filtering:** The training times for the filtered-in FT(i) are relatively much larger than LIG(i) as shown in Figure 9, Figure 10 and Figure 11. However, the total execution times for ITO included training and parameter-tuning for SVM and DNN as well which may have been filtered-out for Datasets A and B. For example, for Dataset C, SVM training times fell around 3000 s to 4000 s (1.1 h each) while DNN training times fell around 30,000 s to 35,000 s (8.3–9.7 each).

## 9. Conclusions and Future Directions

The scarcity of samples, digitization errors, and curse-of-dimensionality of microarray data makes it hard to reliably and accurately classify cancerous cells and avoid overfitting. A number of FSS and classification techniques have been applied to this domain to produce higher accuracies; however, there is still room for more improvement on reliability and generalization of these techniques. The curse of dimensionality and data scarcity can be addressed through the use of heterogeneous models built from subsets of data.

This paper showed that, regardless of the dataset, the accuracy and reliability of a model is inversely proportional (Figure 3a–c and Figure 1a–c) and hence both these factors should be considered when evaluating a model. A notion of efficiency index ρ is introduced which can be used as a single, more dependable factor to choose the best model amongst the available choices. The ITO algorithm introduced in this paper enhances the efficiency index of the underlying LIG and FT models as shown in Table 7, Table 8 and Table 9 and produces an HAHR classification model. The proposed algorithm is a generalized approach which balances the exploration through LIG and exploitation through FT to find a promising initial baseline and optimizes the results beyond this baseline. It leaves the choice of underlying LIG and FT members open to the user. A more advanced LIG or FT selection can further enhance the optimality of the overall model. Further study can be conducted to apply the proposed algorithm on datasets other than MAQC-II for wider comparisons.

For the LIG members, both majority-voting and soft-ensembles produced the same results. However, it is because the underlying classifiers return the predicted class labels instead of raw prediction values. It would be interesting to measure the impact of replacing the predicted class labels with the raw prediction values for soft ensembles. The advantage of soft ensembles was evident when used for FT members. Another future direction can be to cluster the erroneous instances separately and construct a focused model for those hard instances. Once a subset is trained on this cluster, it can be added to the beginning of the classification pipeline to bifurcate the instances accordingly. Use of GPUs/parallel computing for FSS generation and classification should be explored to reduce the overall execution time. Finally, the use of LIG as a filtering step for FT attack vectors should also be explored as potential areas of improvements for the ITO Algorithm.

## Figures and Tables

**Table 1:** MAQC-II datasets available at https://www.ncbi.nlm.nih.gov/geo/query/acc.cgi.

Dataset	Endpoint	Accession Code	Features	Training Samples			Validation Samples		
				+ve	−ve	Total	+ve	−ve	Total
Dataset A	Hamner	GSE24061	1004004	26	44	70	28	60	88
Dataset B	Iconix	GSE24417	0010560	73	143	216	57	144	201
Dataset C	NIEHS	GSE24363	0695556	79	135	214	78	126	204

**Table 2:** ϵ Thresholds.

Dataset	$\epsilon_{\mathrm{LIG}}$	$\epsilon_{\mathrm{FT}}$
A	0.639793072	0.4923244337
B	0.1025047719	0.09680510363
C	0.6312148229	0.6648090879

**Table 3:** Improvements by $LIG_{Ensemble}$.

		LIG(i)		${\mathrm{LIG}}_{\mathrm{Ensemble}}$	
Dataset	|LIG|	Accuracy	MCC	Accuracy	MCC
A	98	75%	0.89	**95%**	**0.90**
B	07	69%	**0.18**	**72%**	0.21
C	80	88%	0.76	**90%**	**0.82**

**Table 4:** Improvements by $FT_{Ensemble}$.

		FT(i)		${\mathrm{FT}}_{\mathrm{Ensemble}}$	
Dataset	|FT|	Accuracy	MCC	Accuracy	MCC
A	199	70%	0.82	**97%**	**0.92**
B	12	68%	**0.16**	**72%**	0.08
C	108	90%	0.81	**91%**	**0.84**

**Table 5:** All Datasets—Accuracy, MCC, and ρ.

Dataset	Measure			Ensembles			
		LIG(i)	FT(i)	LIG	FT	LIG & FT Combined	ITO Tuned
		(X1)	(X2)	(X3)	(X4)	(X5)	(X6)
A	Accuracy	0.75	0.70	0.95	0.97	0.98	**0.99**
	MCC	0.89	0.82	0.90	0.92	0.95	**0.97**
	Efficiency (ρ)	0.76	0.69	0.86	0.89	0.93	**0.96**
B	Accuracy	0.69	0.68	0.71	0.72	0.71	**0.75**
	MCC	0.18	0.16	0.06	0.08	0.00	**0.33**
	Efficiency (ρ)	0.15	0.14	0.05	0.06	0.00	**0.24**
C	Accuracy	0.88	0.90	0.90	0.91	0.90	**0.92**
	MCC	0.76	0.81	0.82	0.84	0.83	**0.87**
	Efficiency (ρ)	0.74	0.76	0.74	0.76	0.75	**0.80**

**Table 6:** All Datasets—ITO Improvement %age over LIG(i), FT(i), LIG Ensemble, FT Ensemble, and Combined Ensemble.

Dataset	Measure	ITO Improvement % Age Over				
		LIG	FT	LIG Ensemble	FT Ensemble	LIG & FT Combined Ensemble
		(X6−X1)/X6%	(X6−X2)/X6%	(X6−X3)/X6%	(X6−X4)/X6%	(X6−X5)/X6%
A	Accuracy	24.24%	29.29%	04.04%	02.02%	01.01%
	MCC	08.25%	15.46%	07.22%	05.15%	02.06%
	Efficiency (ρ)	20.83%	28.12%	10.42%	07.29%	03.12%
B	Accuracy	08.00%	09.33%	05.33%	04.00%	05.33%
	MCC	45.45%	51.52%	81.82%	75.76%	100%
	Efficiency (ρ)	37.5 0%	41.66%	79.17%	75.00%	100%
C	Accuracy	04.35%	02.17%	02.17%	01.07%	02.17%
	MCC	12.64%	06.90%	05.75%	03.45%	04.60%
	Efficiency (ρ)	07.50%	05.00%	07.50%	05.00%	06.25%

**Table 7:** Comparison with Results reported in Literature for an MAQC-II Dataset A.

Method	MCC	Accuracy
MAQC-II [12]	0.210	-
AID [56]	0.293	-
Kun [56]	0.407	-
Kun${}_{tie}$ [56]	0.303	-
Kun${}_{genes}$ [56]	0.346	-
EJLR [57]	0.57	-
Monte Carlo simulation as reported in [36]	0.270	67.3%
ITO algorithm	**0.950**	**98%**

**Table 8:** Comparison with Results reported in Literature for MAQC-II Dataset C.

Method	MCC	Accuracy
MAQC-II [12]	0.830	-
AID [56]	0.793	-
Kun [56]	0.812	-
Kun${}_{tie}$ [56]	0.804	-
Kun${}_{genes}$ [56]	0.781	-
Kun${}_{all}$ [56]	0.792	-
t-test with KNN (Mean Centering) [57]	0.80	-
Monte Carlo simulation as reported in [58]	0.795	90.25%
ITO algorithm	**0.870**	**92%**

**Table 9:** Comparison with Results reported in Literature for MAQC-II Dataset B.

Method	MCC	Accuracy
MAQC-II [12]	0.42	-
Ratio-G [57]	**0.50**	-
ITO algorithm	0.33	**75%**

**Table 10:** Min and Max times for FSS Generation for Datasets A, B, and C.

Data Set	Number of Features	Min Time for FSS (Size 10)	Max Time for FSS (Size 250)
A	1,004,004	26 h	>72 h
B	10,560	50 min	2.6 h
C	695,556	24 h	72 h

## Abbreviations

The following abbreviations are used in this manuscript:

ITO	Infiltration Tactics Optimization algorithm.
MAQC-II	Microarray Quality Control study Phase II
mRMR	Minimum Redundancy Maximum Relevance.
MCC	Matthews Correlation Coefficient
HAHR	High Accuracy, High Reliability
